# New insights into cochlear sound encoding

**DOI:** 10.12688/f1000research.8924.1

**Published:** 2016-08-26

**Authors:** Tobias Moser, Christian Vogl

**Affiliations:** 1 Institute for Auditory Neuroscience and InnerEarLab, University Medical Center Göttingen, Göttingen, Germany; 2 Auditory Neuroscience Group, Max-Planck-Institute for Experimental Medicine, Göttingen, Germany; 3 Synaptic Nanophysiology Group, Max-Planck-Institute for Biophysical Chemistry, Göttingen, Germany; 4 Auditory Neuroscience and Optogenetics Group, German Primate Center, Göttingen, Germany

**Keywords:** inner hair cell, synaptic ribbon, otoferlin

## Abstract

The inner ear uses specialized synapses to indefatigably transmit sound information from hair cells to spiral ganglion neurons at high rates with submillisecond precision. The emerging view is that hair cell synapses achieve their demanding function by employing an unconventional presynaptic molecular composition. Hair cell active zones hold the synaptic ribbon, an electron-dense projection made primarily of RIBEYE, which tethers a halo of synaptic vesicles and is thought to enable a large readily releasable pool of vesicles and to contribute to its rapid replenishment. Another important presynaptic player is otoferlin, coded by a deafness gene, which assumes a multi-faceted role in vesicular exocytosis and, when disrupted, causes auditory synaptopathy. A functional peculiarity of hair cell synapses is the massive heterogeneity in the sizes and shapes of excitatory postsynaptic currents. Currently, there is controversy as to whether this reflects multiquantal release with a variable extent of synchronization or uniquantal release through a dynamic fusion pore. Another important question in the field has been the precise mechanisms of coupling presynaptic Ca ^2+^ channels and vesicular Ca ^2+^ sensors. This commentary provides an update on the current understanding of sound encoding in the cochlea with a focus on presynaptic mechanisms.

## Introduction

In the mammalian cochlea, inner hair cells (IHCs)—the genuine sensory cells of the cochlea transform sound-induced mechanical signals into a neural code at their ribbon synapses. Upon hair bundle deflection, mechanotransducer channels, located in the stereociliar tips, provide hair cell depolarization. This process leads to presynaptic glutamate release from IHCs onto spiral ganglion neurons (SGNs), and ultimately activates the auditory pathway. Coding of sound at IHC ribbon synapses achieves impressive performance: each glutamatergic presynaptic active zone (AZ) of an IHC provides the sole excitatory input to a postsynaptic SGN. Yet, each single AZ drives SGN spike rates at sound onset in the kilohertz range and supports firing at hundreds of hertz during ongoing stimulation. Moreover, these synapses are capable of transmitting information on the timing of the stimulus with submillisecond precision.

The underlying mechanisms that mediate this performance have remained enigmatic but likely relate to the molecular and structural specializations of the IHC ribbon-type AZ. Here, we briefly review the latest progress on the molecular anatomy and physiology of the IHC ribbon synapse, with a focus on the presynaptic AZ (for recent reviews of the postsynaptic SGN, see 1, 2.) Dysfunction or loss of IHC synapses causes a specific form of sensorineural hearing impairment: auditory synaptopathy (recently reviewed in 3). We will then summarize recent experimental and theoretical work that has corroborated the Ca ^2+^ nanodomain hypothesis of Ca ^2+^ influx-exocytosis coupling at IHC ribbon synapses. Finally, we will provide a brief overview of the current state of the debate on the mode of exocytosis at the hair cell AZ, which remains a hot topic of current research.

## Unconventional presynaptic molecular composition

**Figure 1.**
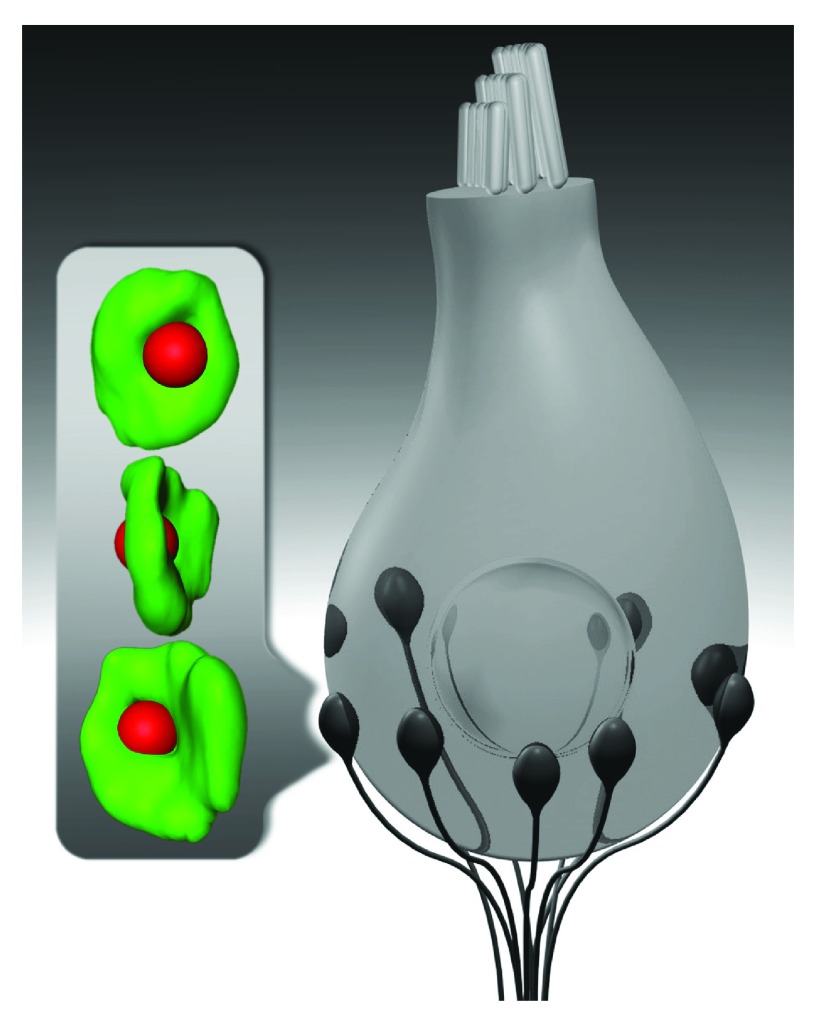
Inner hair cells drive sound encoding in several spiral ganglion neurons. Schematic drawing of an inner hair cell (gray) and its synapses with spiral ganglion neurons (black). Inset shows super-resolution (4Pi) images of an immunolabeled inner hair cell synapse with the synaptic ribbon (red) placed opposite to the center of the postsynaptic AMPA receptor cluster (green). Each spiral ganglion neuron is thought to receive input from one ribbon-type inner hair cell active zone at the postsynaptic swelling of its peripheral neurite.

The synaptic ribbon represents the most prominent structural deviation from “conventional” glutamatergic synapses of the vertebrate central nervous system ( Figure 1). Depending on the cell type, developmental stage, and animal species under investigation, the ribbon can assume various shapes and sizes ^4– 6^. The main structural component of synaptic ribbons is RIBEYE ^7^, a protein assembled from an aggregation-prone A domain and an enzymatically active B domain, both of which are transcribed from the *CtBP2* gene ^8^. The synaptic ribbons help cluster large complements of Ca ^2+^ channels and readily releasable vesicles at the IHC AZ, thereby enabling synchronous auditory signaling and also promoting continuous vesicle replenishment ^9– 11^. Ribbons are also critical for sensory processing in the retina, where they serve similar functions ^12– 14^, and, in addition, seem to play a role in coupling Ca ^2+^ channels to release sites ^15^.

In addition to unexpectedly finding that IHC ribbon synapses appear to operate without neuronal SNAREs ^16^ and the classic neuronal Ca ^2+^ sensors synaptotagmin 1 and 2 ^17, 18^, we have recently come to realize that SNARE regulators such as complexins ^19, 20^, as well as priming factors of the Munc13 and CAPS families ^21^ which are critical for transmission at many synapses, also seem to be missing from IHCs. Instead, hair cells employ the multi-C _2_-domain protein otoferlin ^22^, a member of the ferlin family of membrane fusion-related proteins (reviewed in 23, 24), which is a tail-anchored protein and requires the TRC40 pathway for efficient targeting to the endoplasmic reticulum ^25^. Otoferlin clusters below the synaptic ribbon ^21^ and seems to assume multiple roles in hair cell exocytosis. For example, otoferlin has been suggested (i) to act as the putative Ca ^2+^ sensor in IHCs ^22, 26^, (ii) to facilitate vesicular priming and replenishment ^21, 27^, and (iii) to participate in exocytosis-endocytosis coupling through direct interaction with the adaptor protein 2 (AP-2) complex ^28, 29^. It is tempting to speculate that IHCs evolved this unconventional molecular machinery in order to achieve the utmost performance. One possible requirement could be a rapid and low-affinity engagement of synaptic vesicles with release sites with molecular links to a nearby Ca ^2+^ channel, followed by rapid clearance of vesicular lipid and proteins from that site for it to be quickly reloaded. Clearly, more work is required to elucidate the molecular fusion machinery of IHCs.

Besides the presence of otoferlin, IHC AZs are characterized by large Ca ^2+^ channel clusters, which localize underneath the presynaptic density ^30^ and consist predominantly of pore-forming L-type Ca _V_1.3 subunits ^31, 32^, auxiliary Ca _V_β2 ^33^, and likely yet-to-be-identified Ca _V_α2δ subunits ^34^. Ca ^2+^ channel clustering depends on multiple molecular scaffolds, such as Bassoon or the ribbon (or both) ^10, 35^ as well as RIM2α and β ^36^. The seamless interplay and correct localization of these proteins is not only required for establishing a normal Ca ^2+^ channel complement ^10, 36^ but also critical to stabilize a large readily releasable pool of vesicles at the AZ ^10, 11, 36^. Moreover, IHC AZs contain additional scaffolds such as Piccolino, a short splice variant of Piccolo ^37^, and the Usher protein harmonin that directly interacts with presynaptic Ca ^2+^ channels, regulates their gating, and likely promotes their proteasomal degradation ^38, 39^. Although the endocytic machinery is still largely uncharted, it was recently shown to include AP-2 ^28, 29^, dynamins ^40, 41^, amphiphysin, and clathrin heavy chain ^41^.

## Tight spatial coupling of Ca ^2+^ channels and vesicular Ca ^2+^ sensors

The manner in which Ca ^2+^ influx couples to vesicle fusion critically determines the properties of synaptic transmission. Two limiting cases can be distinguished (reviewed recently in 42– 44): (i) “pure” Ca ^2+^ nanodomain control, in which the Ca ^2+^ driving the fusion of a given vesicle is contributed by an individual voltage-gated Ca ^2+^ channel, and (ii) “pure” Ca ^2+^ microdomain control, where the amount of Ca ^2+^ at the Ca ^2+^ sensor is governed by a population of Ca ^2+^ channels, with negligible impact of individual channels. Aside from the precise topography of the channels with respect to the vesicular Ca ^2+^ sensors and their numbers and open probabilities, the Ca ^2+^-binding properties of the vesicular Ca ^2+^ sensor and the cytosolic Ca ^2+^ buffering at the AZ govern the coupling ^45^. Previous work has examined the Ca ^2+^-binding properties of the Ca ^2+ ^ sensor of fusion by combining whole-cell Ca ^2+^ uncaging and membrane capacitance measurements in IHCs of mice right after hearing onset ^46^. In these experiments, a requirement for four to five Ca ^2+^ ions to bind cooperatively prior to fusion was indicated, and an overall low Ca ^2+^ affinity of the sensor can be assumed. Note however, that this approach yielded massive exocytosis (added membrane equivalent to 15% of the cell’s surface). Hence, it is unlikely to be entirely mediated by exocytosis at IHC AZs, but probably also involved extrasynaptic exocytosis. This calls for revisiting the intrinsic Ca ^2+^ dependence of synaptic vesicle fusion by using refined approaches to exocytosis at AZs, ideally of more mature IHCs.

Classic ^47^ and more recent ^30, 31, 48^ work indicates that hair cell AZs harbor tens of Ca ^2+^ channels on average. However, within a given IHC, regardless of its tonotopic position, the number of Ca ^2+^ channels per AZ varies dramatically. This presynaptic heterogeneity is thought to be related to the requirements of wide dynamic range sound encoding ^30, 49– 51^. Interestingly, IHCs display opposing gradients of their AZs for Ca ^2+^-channel complement (higher at the modiolar side, facing the ganglion) and voltage-dependence of activation (voltage for half-maximal activation more hyperpolarized at the pillar side, facing away from the ganglion) ^52^.

Moreover, depending on the experimental strategy, estimates for the maximal Ca ^2+^ channel open probability at IHC AZs vary between 0.2 ^48^ and 0.4 ^30^. To date, the exact topography of individual Ca ^2+^ channels within the observed stripe-like clusters beneath the ribbon and their putative molecular linkers to vesicular release sites remains to be experimentally determined. Here, biophysically constrained modeling has proven to be a useful tool in exploring the consequences and feasibility of various scenarios (see below, 30, 53). Moreover, another interesting aspect in this context will be the detailed identification of the molecular processes governing AZ maturation, in particular, in regard to the progressive tightening of Ca ^2+^ channel-synaptic vesicle coupling during early postnatal development ^30, 48, 54^.

Endogenous Ca ^2+^ buffering has been studied in hair cells of various species ^53, 55– 58^ typically revealing substantial concentrations of Ca ^2+^-binding sites (up to a few millimolar). Recently, measurements of exocytic membrane capacitance changes in mutant IHCs lacking the three major cytosolic EF-hand Ca ^2+^-binding proteins (that is, calbindin-28k, calretinin, and parvalbumin) were combined with Ca ^2+^ buffer substitution—using different concentrations of synthetic Ca ^2+^ chelators with either slow (EGTA) or fast (BAPTA) kinetics—and computational modeling of stimulus-secretion coupling ^53^. With this combinatorial approach, the effective average coupling distance between the Ca ^2+ ^sensor of the release site and the nearest Ca ^2+^ channels was estimated to equate to approximately 17 nm in mature IHCs and this is well in line with a Ca ^2+^ nanodomain-like control of exocytosis and similar to previous estimates (approximately 23 nm; ^59^).

The notion of a Ca ^2+^ nanodomain-like control of exocytosis is supported by observations of a lower apparent Ca ^2+^ cooperativity (approximately 1.4) of IHC exocytosis upon changes in the channel open probability, when compared with that found with changes in single-channel Ca ^2+^ current (3–4; ^30, 31, 59^). The interpretation of these discrepant apparent cooperativity estimates as evidence for Ca ^2+^ nanodomain-like control of exocytosis was further substantiated by modeling ^30^. There, 50 Ca ^2+^ channels were distributed over an area of 80 × 400 nm, aiming to match the Ca ^2+^-channel clusters, assuming different topographies of the Ca ^2+ ^channels to a dozen release sites that were placed at the rim of the Ca ^2+^ channel cluster. Modeling of Ca ^2+^-triggered exocytosis was constrained by experimental observations as much as possible. When channels were randomly positioned, the apparent Ca ^2+^ cooperativity of exocytosis during changes in the number of Ca ^2+ ^channels was close to two, and hence higher than the experimentally observed value of 1.4. The physiological Ca ^2+^ cooperativity was best matched when allocating one molecularly coupled channel to each release site, while the other channels were distributed randomly but respected an exclusion zone of 10 nm around the coupled channels. This was taken to support the notion of Ca ^2+^ nanodomain-like control of exocytosis at the IHC AZs of mice after hearing onset, likely realized by molecular coupling of a “private” channel to the release site.

During development, Ca ^2+^ influx-exocytosis coupling tightens from Ca ^2+^ microdomain-like control to Ca ^2+^ nanodomain-like control ^30^, as also found for other synapses ^60^. At this point, Ca ^2+^ microdomain control of exocytosis seems less likely for mature IHC synapses. Initially, this mechanism had been considered as an explanation for the low apparent Ca ^2+^ cooperativity of exocytosis in mature IHCs, which was thought to employ a linear Ca ^2+^ sensor (one Ca ^2+^-binding step by synaptotagmin 4) after the onset of hearing ^61^. Alternatively, a lower cooperativity, even in the presence of a sensor with several Ca ^2+^-binding steps, was suggested to result in a linear apparent Ca ^2+^ dependence in whole-cell membrane capacitance measurements, due to summing exocytosis from heterogeneous, but Ca ^2+^ microdomain-governed AZs in IHC capacitance measurements of exocytosis ^62^. However, whereas the former hypothesis seems hard to reconcile with the comparable and supralinear intrinsic Ca ^2+^ dependence of fusion prior and after hearing onset ^30^, the latter appears incompatible with the indications for Ca ^2+^ nanodomain control of exocytosis at the single-synapse level ^59^.

Future experiments should elucidate the topography and mobility of individual Ca ^2+^ channels within the cluster and dissect putative linker proteins connecting release sites to the nearest Ca ^2+^ channel(s). Moreover, further characterization of the molecular composition of the Ca _V_1.3 Ca ^2+^ channel complexes, also testing the presence and role of splice variants of the pore-forming Ca _V_1.3α subunit, will assist in understanding (i) the molecular mechanisms that govern the heterogeneity of Ca ^2+^ channel expression, (ii) the developmental tightening of excitation-secretion coupling, and (iii) the respective contributions of individual Ca ^2+^ channel variants to shaping the efficiency of presynaptic Ca ^2+^ influx at individual IHC AZs.

## Large and variable excitatory postsynaptic currents: the uniquantal versus multiquantal release debate

We will now focus on the mode of vesicular release at auditory ribbon synapses, a mechanism that remains only partially understood. The phenomena that raised uncertainty about this fundamental process of synaptic transmission are (i) a remarkable heterogeneity of AMPA receptor-mediated excitatory postsynaptic current (EPSC) amplitudes, that can range from about 20 to more than 800 pA at postsynaptic SGN terminals of rodents and (ii) the differences in release kinetics, as reflected in variable rise times and waveforms of the EPSCs ^63^.

Conventionally, quanta of neurotransmitter are released spontaneously (“uniquantal release”), thereby producing so-called miniature EPSCs (mEPSCs). These mEPSCs are uniform in size and have characteristic monoexponentially decaying waveforms. Such mEPSCs are thought to correspond to spontaneous and statistically independent fusion of individual vesicles, constituting the basis of the quantal hypothesis of transmitter release ^64^. At IHC synapses, large variance of EPSC amplitudes and waveforms is found in individual postsynaptic boutons even in complete absence of stimulation. Synchronized (statistically dependent) release of multiple vesicles (“synchronized multiquantal release”) was offered as a plausible mechanism explaining such EPSC heterogeneity ^59, 63, 65^. Synchronized multiquantal release has also been indicated for hair cell synapses of turtle and bullfrog papillae ^66– 69^, and for other sensory synapses, such as those in retinal bipolar cells ^70– 72^.

Heterogeneity of the EPSC shape might result from varying degrees of synchronicity of multiquantal release ^63, 73^. Potential mechanisms mediating synchronized multiquantal release include release site synchronization and compound or cumulative fusion of vesicles (reviewed in 2, 4, 74). The synaptic ribbon might contribute to synchronizing multiquantal release by clustering presynaptic Ca ^2+^ channels and tethering a large complement of release-ready synaptic vesicles ^10, 21, 36, 71, 75^. One thought is that Ca ^2+^ influx through an individual Ca ^2+ ^channel could synchronously trigger the fusion of several nearby vesicles underneath the synaptic ribbon ^66^. Alternatively, vesicles might pre-fuse to each other to form larger quanta prior to fusion to the plasma membrane (compound exocytosis), or fuse to a vesicle that is in the process of release (cumulative exocytosis).

Recently, uniquantal release through a dynamic fusion pore has been suggested as an alternative hypothesis for IHC synapses of rodents ^76^. The motivation came from considering the spike rates of hundreds of hertz over prolonged periods of time, which, according to the multiquantal hypothesis (on average, six vesicles per EPSC ^59^), would require at least sixfold-higher vesicle release rates, which seem very high considering measured rates of sustained exocytosis of about 700 vesicles per second ^27^. Moreover, in conditions omitting the synchronizing effect of presynaptic Ca ^2+^ entry, large monophasic, as well as multiphasic, EPSCs persisted ^76^, seemingly arguing against a Ca ^2+^-synchronized multiquantal release scenario at the IHC ribbon synapse. In addition, biophysically constrained mathematical modeling of compound exocytosis suggested the presence of large vesicles near the AZ membrane, which was not found in electron microscopy of stimulated samples. These latter findings are difficult to reconcile with a synchronized multiquantal release mode to take place at mammalian IHC ribbon synapses. So, could a uniquantal release model offer an explanation for the large heterogeneity of EPSC amplitudes?

Uniquantal release through a dynamic fusion pore has also been proposed for other synapses ^77, 78^; however, for this scenario to be plausible at IHC synapses, two major questions arise: (i) could the glutamate content of a single synaptic vesicle elicit the observed large EPSCs (on average, about 300 pA) and (ii) could transmitter release be governed by a fusion pore that might regulate the extent and timing of release events? Using mathematical modeling—constrained by morphological estimates of the postsynaptic AMPA receptor clusters ( Figure 1) and assumptions on glutamate content and AMPA receptor density and function—the study concluded that single-vesicle release might suffice to trigger large-amplitude EPSCs ^76^. Moreover, model prediction and deconvolution of EPSCs suggested that fusion pore regulation could account for the observed variable EPSC shapes. In detail, the authors suggested a model in which there is either immediate and full collapse upon vesicle fusion (potentially explaining monophasic EPSCs) or alternatively the formation of a transitory instable fusion pore prior to collapse, potentially flickering open and closed—a mechanism permitting progressive glutamate unloading that may explain the observation of multiphasic EPSCs. In addition, variable vesicle size that occurs also in the absence of homotypic vesicle-to-vesicle fusion and different filling states of vesicles ^79^ might contribute to the EPSC heterogeneity at IHC synapses. Finally, other mechanisms such as spill-over of glutamate from neighboring synapses and postsynaptic receptor properties need to be considered when relying on postsynaptic recordings.

These different hypotheses might not be mutually exclusive, and appear to strongly depend on the chosen experimental model system. Further experiments, such as (i) membrane capacitance recordings of individual fusion events, (ii) electron tomography of synapses immobilized within milliseconds after stimulation, or (iii) super-resolution fluorescence live-cell imaging of vesicular exocytosis, will help in the future to pinpoint the mode of exocytosis at hair cell ribbon synapses.
